# Diversity of larval habitats of *Anopheles* mosquitoes in urban areas of Benin and influence of their physicochemical and bacteriological characteristics on larval density

**DOI:** 10.1186/s13071-022-05323-6

**Published:** 2022-06-13

**Authors:** Donald Hessou-Djossou, Innocent Djègbè, Koffi Mensah Ahadji-Dabla, Odilon M. Nonfodji, Geneviève Tchigossou, Rousseau Djouaka, Sylvie Cornelie, Luc Djogbenou, Martin Akogbeto, Fabrice Chandre

**Affiliations:** 1 Département des Sciences de la Vie et de la Terre, Ecole Normale Supérieure de Natitingou, Université Nationale des Sciences, Technologie, Ingénierie et Mathématiques (UNSTIM), Natitingou, Bénin; 2 Plateforme Agriculture Environnement Santé, Institut International d’Agriculture Tropicale (IITA-Bénin), Cotonou, Bénin; 3 Laboratoire d’Ecologie et d’Ecotoxicologie (LaEE), Département de Zoologie, Faculté des Sciences, Université de Lomé, Lomé, Togo; 4 Laboratoire de Chimie de l’Eau et de l’Environnement (LCEE), Ecole Normale Supérieure de Natitingou, Université Nationale des Sciences, Technologie, Ingénierie et Mathématiques (UNSTIM), Natitingou, Bénin; 5 Maladies Infectieuses et Vecteurs: Ecologie, Génétique, Evolution et Contrôle (MIVEGEC), UMR IRD 224-CNRS 5290-Université de Montpellier 2, Montpellier Cedex 5, France; 6 Institut Régional de Santé Publique (IRSP), Université d’Abomey-Calavi (UAC), Ouidah, Bénin; 7 Centre de Recherche Entomologique de Cotonou (CREC), Ministère de la Santé, Cotonou, Bénin

**Keywords:** Ecology, Physicochemical parameters, Faecal coliforms, Larval density, *Anopheles*

## Abstract

**Background:**

The implementation of anti-larval strategies in the fight against malaria vectors requires fundamental knowledge of their oviposition sites. The aim of this study was to assess the spatial and temporal distribution of *Anopheles* breeding sites as well as the influence of abiotic and biotic factors on the proliferation of larvae in urban and non-urban areas of Benin.

**Methods:**

Sampling of *Anopheles* larvae was carried out during the rainy and dry seasons in urbanized and non-urbanized areas of the cities of Cotonou, Bohicon, Parakou, and Natitingou in Benin. The *Anopheles* larval breeding sites were georeferenced and characterized by their nature, type, physicochemical (pH, temperature, dissolved oxygen, conductivity, turbidity, salinity) and biological attributes (larval density and coliform density).

**Results:**

A total of 198 positive breeding sites for *Anopheles* larvae were identified, comprising 163 (82.3%) in the rainy season and 35 (17.7%) in the dry season. Out of these larval habitats, 61.9% were located in urbanized areas, and were predominantly puddles. Principal component analysis revealed a high positive correlation of larval density with temperature and dissolved oxygen, and with salinity in the coastal zone. In addition, cross-sectional analysis of the microbiological results with larval density showed a significant negative correlation between larval productivity and faecal coliform load.

**Conclusions:**

This study indicated the presence of multiple larval habitats of *Anopheles* in the urban areas which were created through human activities, and associations between larval density and intrinsic factors of the habitats such as temperature, dissolved oxygen and faecal coliform load. This type of information may be useful for the implementation of appropriate control strategies in urban areas, including regulation of the human activities that lead to the creation of breeding sites, proper environmental management and targeted larvicidal use.

**Graphical abstract:**

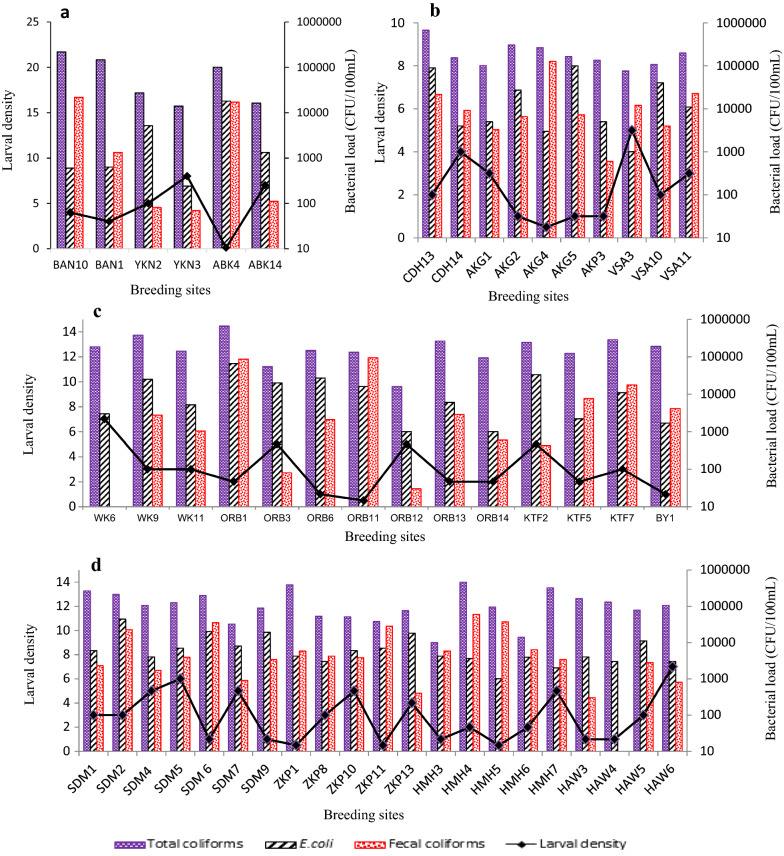

## Background

In recent years there has been renewed interest in urban malaria, particularly in sub-Saharan Africa. It is estimated that 24.8–103.2 million clinical episodes of malaria occur annually in urban areas that are endemic for malaria [1]. Other studies have estimated that nearly 150 million city dwellers are currently at risk of malaria and that 6–28% of annual malaria cases in Africa occur in urban areas [2, 3]. Urbanization is thought to lead to the improvement of infrastructure, better-quality ‘mosquito-proof’ housing, an increase in access to healthcare, and a reduction in vector breeding sites. Malaria vector species are known to prefer clean water for breeding, which is difficult to access in polluted urban areas; a higher ratio of humans to mosquitoes is also thought to lead to a decreased human biting rate [4]. However, despite these encouraging factors, malaria transmission persists in African cities and, in some cases, at even higher levels than in surrounding areas [5]. Indeed, some African cities presently experience entomological inoculation rates exceeding 80 infective bites per person per year [6].

In Cotonou, the economic capital of Benin, malaria transmission is perennial, and the spleen rates are between 40 and 60%, indicating that this is a meso- to hyperendemic area where malaria infection and disease represent substantial burdens for the local population [7]. These figures might be revised upwards due to the galloping demographic growth of the continent of Africa and mass migration to urban centres. According to recent projections, more than 60% of the African population will live in urban areas by 2050 [8]. In addition, the rapid urbanization in large cities is often anarchic and thus beyond political control. This type of urbanization leads to the emergence of new types of lifestyles, which in turn lead to an increase in mosquito larval habitats, specifically those of *Anopheles* species, making these cities important reservoirs of potential vectors [9].

In Benin, as in most sub-Saharan countries, malaria remains a major public health problem, with an increase in malaria incidence and associated mortality, which doubled between 2010 and 2017 [10]. Currently, the two main vector control methods used by the National Malaria Control Program are based on the use of insecticides and target adult mosquitoes through indoor residual spraying (IRS) [11–13] and the use of long-lasting insecticidal nets (LLINs) [14, 15]. However, the development of resistance of *Anopheles* to insecticides, the increasingly exophagous behaviour of *Anopheles*, and the existence of residual transmission when people are not protected by IRS or LLINs, underline the importance of developing complementary methods [15, 16]. Among these, larval control, particularly in urban areas where larval habitats are limited and easily identifiable, has recently been recommended by the World Health Organization [17]. In many countries around the world, larval control through the systematic application of larvicides has been used to complement IRS and LLINs in malaria vector control [18]. Controlling mosquito larval populations is often beneficial because the larvae are usually concentrated, relatively immobile and often easily accessed [19]. The appropriate management of larval habitats could help to suppress vector densities and therefore the transmission of malaria; however, appropriate management requires in-depth and up-to-date knowledge of the spatiotemporal distribution and type of breeding sites colonized by malaria vectors.

Many studies have shown that the establishment of an effective vector control method for mosquitoes must take into account ecoclimatic factors, the bioecology of the vector, the spatiotemporal distribution and physicochemical characteristics of the oviposition sites, and the level of sensitivity of the adult mosquitoes to insecticides [20–22]. Indeed, various factors influence the density and distribution of these mosquito species and their resistance to insecticides. These include climatic factors, plant cover, breeding site types and anthropogenic factors [23]. In addition, the physicochemical (temperature, salinity, hydrogen potential, nitrate levels, etc.) and bacteriological parameters of the breeding sites can also influence the distribution of mosquito species [21, 24]. Such data on mosquito bioecology are lacking for Benin, and this constitutes a serious handicap for the evaluation of vector control strategies there.

The aim of this study was to investigate the distribution of *Anopheles* larvae in four cities of Benin as well as the biotic and abiotic habitat characteristics favourable for their development.

## Methods

### Study areas

**Fig. 1:**
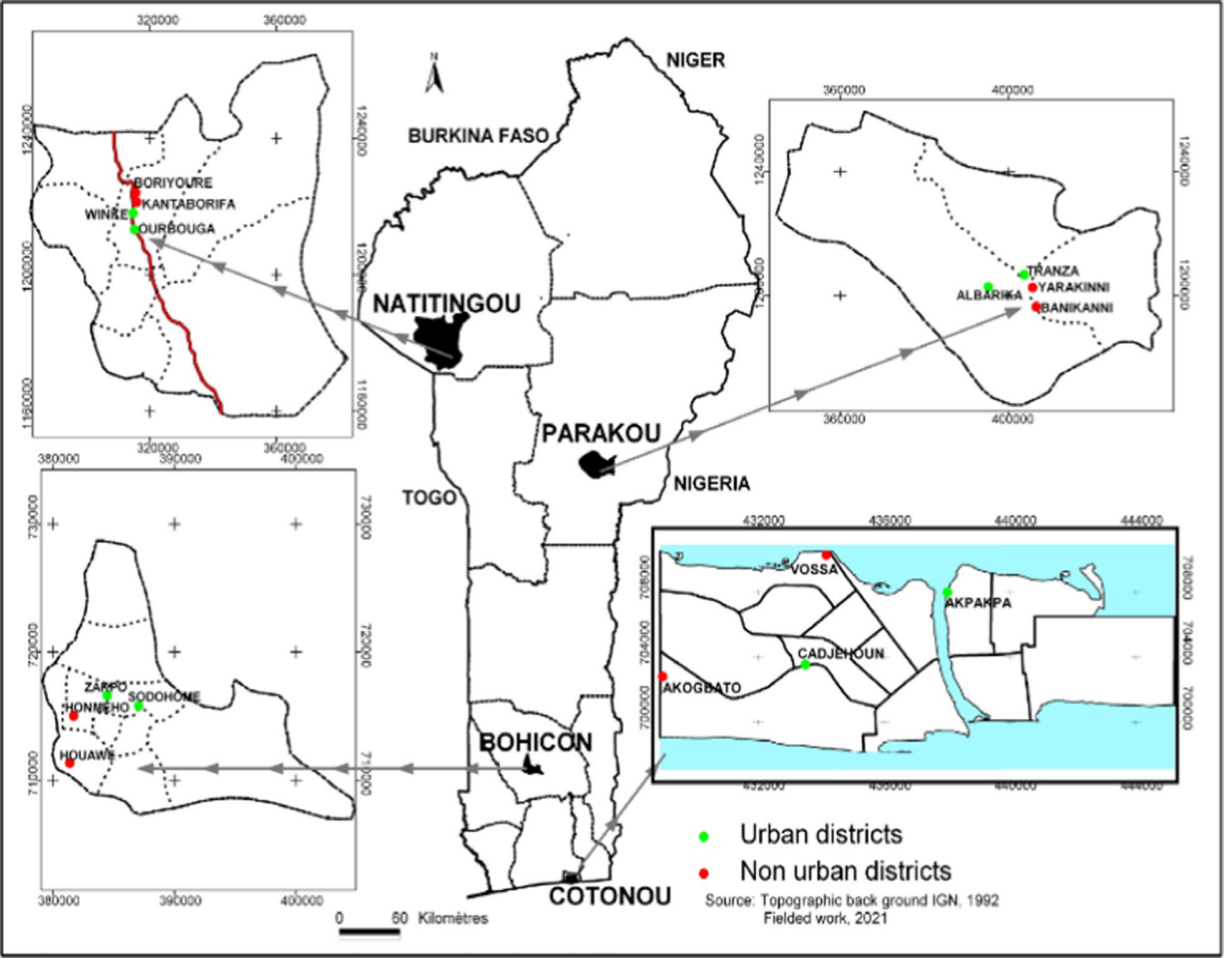
Map of Benin showing the different cities and districts surveyed (insets)

The study took place in four major cities, Cotonou (6°21′55.3″N, 2°25′6″E), Bohicon (7°10′41.7″N, 2°4′0.1″E), Parakou (9°20′13.8″N, 2°37′49.1″E) and Natitingou (10°18′15″N, 1°22′46.6″E), selected along a transect from north to south Benin in order to cover the different ecological zones of the country. Four districts, two urban and two non-urban, were selected in each city (Fig. 1). The urban districts are characterized by planned modern urbanization with houses built on well-demarcated plots. The streets are well laid out and clean. In the non-urban districts, there is no plan of urbanization and the houses are built anarchically, often grouped together, and are often highly insalubrious. These districts have not been divided into demarcated plots and the majority of the people who live in them have poor health and experience low social conditions.

Cotonou is situated within the coastal strip that stretches between Lake Nokoué and the Atlantic Ocean, and is characterized by a typical equatorial climate with two rainy seasons extending from March to June and from September to November. The city of Cotonou covers an area of 79 km^2^ and has a population of around 1,300,000 inhabitants [25]. Rainfall there varies between 900 and 1200 mm, while the average temperature fluctuates between 18 and 35 °C. The landscape of Cotonou is not very rugged and includes swamps and lowlands which are suitable for vegetable gardening. The city has a large number of processing and storage facilities [26].

Bohicon is located in central Benin and is characterized by a transitional subequatorial climate with two rainy seasons (April–June and September–November). It covers an area of 44 km^2^ and has a population of 171,781 inhabitants [25]. The average rainfall is 1025 mm/year and the temperature varies between 25 and 34 °C. The main economic activities are commerce, crafts and agriculture [27].

Parakou is situated in the north-eastern part of the country; it has an average altitude of 350 m above sea level and covers an area of 441 km^2^. Parakou has a population of 254,254 inhabitants [25], and is characterized by the south Sudanese climate (tropical and humid) and one rainy season from May to October. The average annual rainfall is 1200 mm, with most of the rainfall occurring between July and September. The lowest temperatures are recorded in December–January. Manufacturing, commerce and urban agriculture are the main economic activities in Parakou [28].

Natitingou is located in north-western Benin at an altitude of 500 m above sea level, and has a rugged terrain. It covers an area of 3045 km^2^ and has a population of 103,843 inhabitants [25]. The climate is Sudano-Guinean, which is characterized by one rainy season from May to October. The city of Natitingou has high annual rainfall, with up to 1400 mm rainfall in some years. The average temperature is around 27 °C with a range of 17–35 °C during the harmattan. The main economic activities are agriculture, tourism, crafts and commerce [29].

### Larval collection and habitat characterization

Mosquito larvae were collected in the cities of Cotonou, Bohicon, Parakou and Natitingou from August to September 2020 in the rainy season and from January to February 2021 in the dry season to assess the distribution of their breeding sites. The surveys were carrying out from 10 a.m. to 5 p.m. on 3 consecutive days in all the areas of the selected districts.

All open water bodies were considered potential breeding sites and investigated. The presence or absence of larvae was determined after visual inspection of the breeding habitats. Only anopheline larvae and pupae were collected, using the standard dipping techniques as described by Service and Silver [30]. In each breeding site, three to ten dips were carried out, depending on the size of the larval habitat, using a standard 350-mL dipper. After each dip, the contents were transferred to a plastic container and the larval count was estimated [31]. The larvae were then stored in well-labelled plastic containers and transported to the insectarium of the Ecole Normale Supérieure de Natitingou for rearing and morphological identification [32]. Emerging adults from the field larval collections were placed in cages, fed 10% honey solution and kept at 27 ± 2 °C and 72 ± 5% relative humidity.

The breeding sites were categorized as follows: swamps, gutters, pits, puddles or vegetable farms, hoof imprints, tyre tracks, and others. Physical characteristics of the open water collection sites were recorded by the same person in order to maintain consistency in the visual classification. Breeding sites were also classified into two categories after visual inspection: permanent and temporary rain-filled breeding sites.

Measurements of the physicochemical characteristics of the breeding sites were recorded in situ using a Hanna HI 991001 pH meter, a VWR CO300 multiparametric conductivity meter, a WTW OXI 3205 oximeter and a Hanna HI 93703 Turbidity Meter. The following parameters were measured: turbidity [nephelometric turbidity units (NTU)]; pH; temperature (degrees Celsius); conductivity (microsiemens per centimetre); salinity (grams per litre); dissolved oxygen (milligrams per litre); and total dissolved solids (parts per million).

After the measurements had been made, at least 200 mL of water was sampled from each sample breeding site and placed into a sterile flask. The flask was then placed in an icebox and brought back to the laboratory for microbiological analysis of the water.

### Microbiological analysis of water from the breeding sites

The level of microbiological pollution of the breeding sites was evaluated by isolation and then culture of total coliforms and *Escherichia coli* at 37 °C and faecal coliforms at 44 °C. The water was filtered through a 0.45-μm nitrocellulose membrane and the microorganisms cultured on Chromogenic Coliform Agar according to the protocol described by Nonfodji et al. [33].

### Statistical analysis

Pearson’s chi-square test was used to compare the proportions of breeding sites and habitat types between seasons and districts. One-way ANOVA was used to compare larval densities between breeding sites, season and districts. Correlations between the recorded physicochemical variables and larval densities were assessed using Pearson’s correlation analysis and principal component analysis. All the statistical analyses were performed using R software version 4.0.3 (R Development Core Team 2020).

## Results

### Type and distribution of *Anopheles* larval habitats

**Fig. 2:**
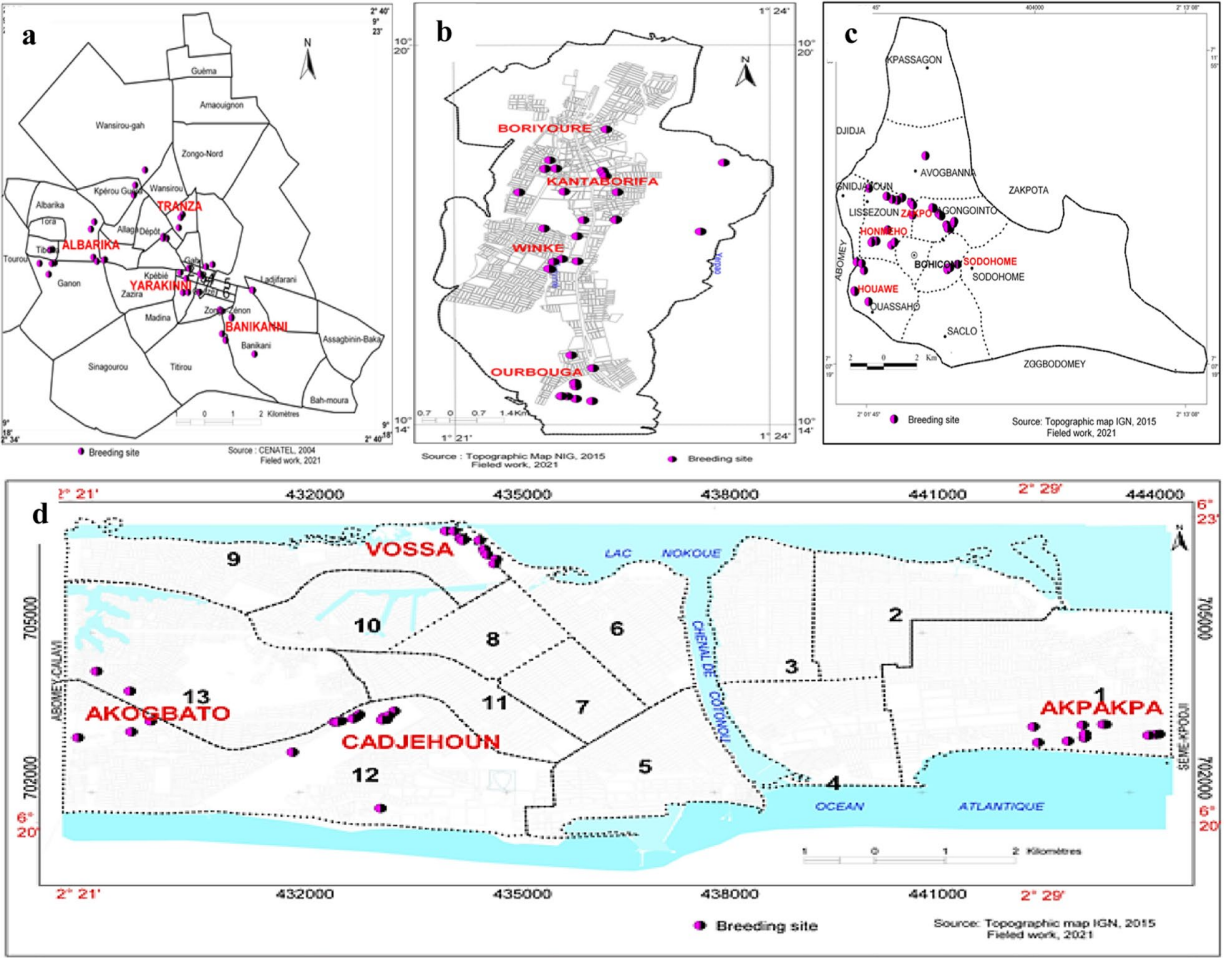
**a**–**d** Spatial distribution of the breeding sites surveyed in the four cities in the rainy season. **a** Parakou, **b** Natitingou, **c** Bohicon, **d** Cotonou

**Fig. 3:**
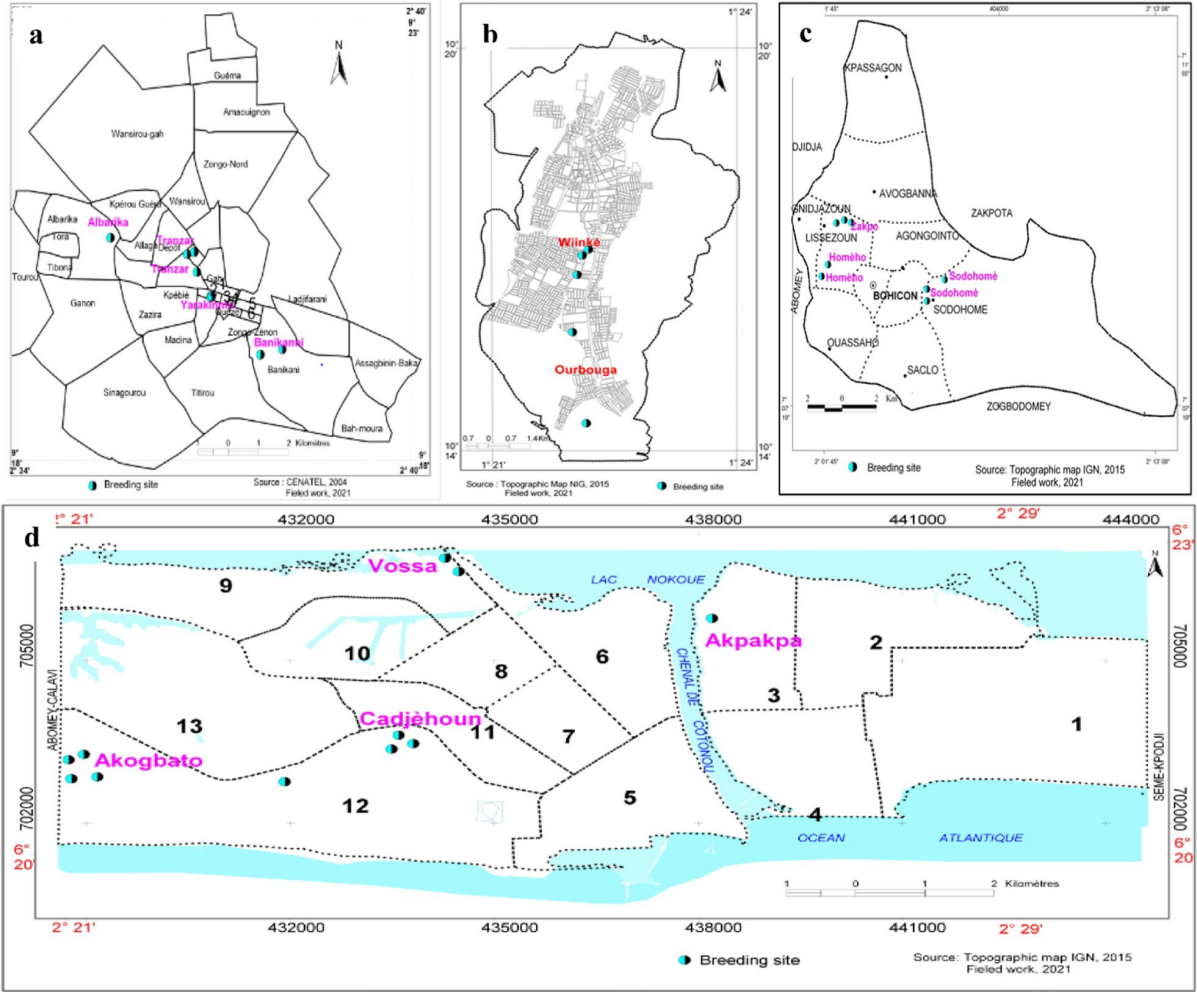
**a**–**d** Spatial distribution of the breeding sites surveyed in the four cities in the dry season. **a** Parakou, **b** Natitingou, **c** Bohicon, **d** Cotonou

Overall, 198 breeding sites positive for *Anopheles* larvae were identified and characterized during the surveys in the four cities; these comprised 163 breeding sites (82.3%) in the rainy season (Fig. 2) and 35 breeding sites (17.7%) in the dry season (Fig. 3). A significant difference was observed in the distribution of *Anopheles* breeding sites according to the seasons (χ^2^ = 58.093, *df* = 1, *P* < 0.0001) (Table 1). During the rainy season, out of 163 breeding sites, 101 (61.9%) were found in urban districts compared to 62 (38.1%) in non-urban districts. In the dry season, 20 (57.1%) and 15 (42.9%) breeding habitats were found in urban and non-urban districts, respectively. No difference was observed in the distribution of breeding sites between urban and non-urban districts according to the collection period. The 198 breeding sites were categorized into 17 types, including puddles (45.9%, *n* = 91), tyres (14.6%, *n* = 29) and gutters (11.1%, *n* = 22), which had the highest frequencies of larvae (Table 2). During the rainy season, puddles were the most frequent habitat type encountered in Natitingou (40%, *n* = 14), Bohicon (67.5%, *n* = 27) and Cotonou (77.8%, *n* = 35), while tyres (32.6%, *n* = 14) and puddles (20.9%, *n* = 9) were the most frequent habitat types encountered in Parakou.

**Table 1:** Distribution of breeding sites in the study sites in the rainy and dry seasons according to the level of urbanization of the districts

Cities	Rainy season					Dry season				
	Urban districts		Non-urban districts		Total	Urban districts		Non-urban districts		Total
	*n*	%	*n*	%	*n* (%)	*n*	%	*n*	%	*n* (%)
Parakou	20	46.5	23	53.5	43 (100)	3	30	7	70	10 (100)
Natitingou	26	74.3	9	25.7	35 (100)	5	100	0	0	5 (100)
Bohicon	27	67.5	13	32.5	40 (100)	6	75	2	25	8 (100)
Cotonou	28	62.2	17	37.8	45 (100)	6	50	6	50	12 (100)
Total	101	61.9	62	38.1	163 (100)	20	57.1	15	42.9	35 (100)

**Table 2:** Types of breeding sites and their mean larval densities in the rainy and dry season according to the level of urbanization of the districts

Seasons	Habitat types	Parakou		Natitingou		Bohicon		Cotonou	
		Urban districts, *n* (mean larval density/dip)	Non-urban districts, *n* (mean larval density/dip)	Urban districts, *n* (mean larval density/dip)	Non-urban districts, *n* (mean larval density/dip)	Urban districts, *n* (mean larval density/dip)	Non-urban districts, *n* (mean larval density/dip)	Urban districts, *n* (mean larval density/dip)	Non-urban districts, *n* (mean larval density/dip)
Rainy season	Gutters	2 (18.5 ± 23.3)	3 (4.3 ± 4.9)	0	1 (5)	5 (11.4 ± 12.0)	1 (1)	0	0
	Tyres	5 (1.5 ± 0.8)	9 (1.9 ± 1.6)	5 (2.5 ± 2.1)	0	4 (2.7 ± 1.7)	2 (3.5 ± 2.1)	1 (2)	0
	Vegetable farms	0	2 (7 ± 4.2)	0	0	0	0	2 (6.5 ± 4.9)	0
	Puddles	2 (1.2 ± 0.3)	7 (4.5 ± 3.5)	10 (3.7 ± 3.1)	4 (5.2 ± 6.5)	17 (3 ± 2.1)	10 (3.4 ± 4.4)	21 (4.3 ± 4.5)	14 (2.5 ± 1.5)
	Tyre tracks	3 (12 ± 12.1)	0	2 (6.5 ± 2.1)	3 (6.3 ± 3.0)	0	0	0	3 (4 ± 3.6)
	Swamps	1 (3)	0	1 (3)	0	0	0	0	0
	Pits	0	0	1 (3)	0	0	0	3 (1.3 ± 0.6)	0
	Water containers for animals	0	1 (1)	0	0	0	0	0	0
	Hoof imprints	1 (2)	0	0	1(2)	0	0	0	0
	Tin can	0	1 (1)	0	0	0	0	0	0
	Ponds	6 (7.2 ± 5.3)	0	5 (6.3 ± 8.3)	0	1 (2)	0	0	0
	Abandoned cans	0	0	1(4)	0	0	0	0	0
	Wells	0	0	1(0.5)	0	0	0	0	0
	Construction sites	0	0	0	0	0	0	1 (6)	0
	Total	20 (8.7 ± 8.9)	23 (3.4 ± 3.2)	26 (3.9 ± 4.3)	9 (5.2 ± 4.9)	27 (4.5 ± 6.0)	13 (3.3 ± 3.9)	28 (4.1 ± 4.2)	17 (2.8 ± 1.9)
Dry season	Gutters	1 (3)	2 (4.5 ± 3.5)	1 (2)	0	2 (4.5 ± 0.7)	2 (4 ± 4.2)	2 (2 ± 1.4)	0
	Tyres	1 (5)	0	0	0	1(2)	0	1(0.5)	0
	Vegetable farms	0	0	2 (4 ± 4.2)	0	1 (5)	0	2 (2.5 ± 0.7)	0
	Puddles	0	2 (2.5 ± 2.1)	2 (1.5 ± 0.70)	0	1(0.1)	0	0	1(2)
	Tyre tracks	0	1 (2)	0	0	0	0	0	1(2)
	Hollow bricks	0	0	0	0	0	0	0	1(0.5)
	Flowerpots	0	0	0	0	0	0	1(1)	0
	Under bridges	0	2 (3.5 ± 3.5)	0	0	1 (2)	0	0	0
	Swamps	0	0	0	0	0	0	0	2 (1 ± 0)
	Pits	1 (0.5)	0	0	0	0	0	0	1 (7)
	Total	3 (2.8 ± 2.2)	7 (3.3 ± 2.4)	5 (2.6 ± 2.1)	0	6 (3.0 ± 1.9)	2 (4 ± 4.2)	6 (1.7 ± 1.1)	6 (2.3 ± 2.4)
*P*-value		0.2667	0.9399	0.5180	**–**	0.5535	0.8180	0.1789	0.1347

In Natitingou, Bohicon and Cotonou, puddles were mostly encountered in urban districts (71.1%, 62.1% and 60%, respectively) rather than in non-urban districts (28.1%, 37.1% and 40%, respectively) during the rainy season (Table 2).

### Larval densities in the breeding sites

The densities of *Anopheles* larvae in each collection period and in the different districts are shown in Table 2. In Parakou, the most productive breeding sites for *Anopheles* larvae were located in the urban districts and included gutters (18.5 ± 23.3 larvae/dip), tyre tracks (12 ± 12.1 larvae/dip) and ponds (7.2 ± 5.3 larvae/dip). Similar observations were made in Natitingou, with tyre tracks (6.5 ± 2.1 larvae/dip) and ponds (5.3 ± 8.3 larvae/dip) the most productive habitats in urban districts. In Bohicon and Cotonou the highest densities of larvae were respectively found in gutters (11.4 ± 12.0 larvae/dip) and vegetable farms (6.5 ± 4.9 larvae/dip) in the urban districts. In the non-urban districts, the highest larval densities were found in vegetable farms in Parakou (7 ± 4.2 larvae/dip) and Cotonou (6.5 ± 4.9 larvae/dip). In Natitingou and Bohicon, the highest larval densities of *Anopheles* were found in tyre tracks in the rainy season (6.5 ± 2.1 larvae/dip) and gutters in the dry season (4 ± 4.2 larvae/dip).

The highest larval densities of *Anopheles* mosquitoes were observed during the rainy season in several districts, but there were no significant differences in the larval densities between the different seasons and between the different districts.

### Nature and types of breeding sites

During the collection period, mosquito larval breeding sites were classified as either temporary or permanent. Temporary sites were mainly rain dependent and dried up when it had not rained for a while. Permanent sites, on the other hand, had a regular source of water, either from a clean water source, or from underground, as was the case for certain low-lying and marshy areas, or from rivers/streams, or from broken pipes.

In the two collection periods, the majority of breeding sites in the four cities were temporary ones. During the rainy season, 81.4% (χ^*2*^ = 11.881, *df* = 1, *P* = 0.0006), 83.3% (χ^*2*^ = 10.742, *df* = 1, *P* = 0.001), 97.5% (χ^*2*^ = 18.501, *df* = 1, *P* < 0.0001) and 93.3% (χ^*2*^ = 18.807, *df* = 1, *P* < 0.0001) of the habitats were temporary in Parakou, Natitingou, Bohicon and Cotonou, respectively (Table 3). During the dry season, the percentages of breeding sites identified as temporary were as follows: 50% in Parakou (χ^*2*^ = 0.0, *df* = 1, *P* = 1.0), 60% in Natitingou (χ^*2*^ = 0.154, *df* = 1, *P* = 0.6949), 100% in Bohicon (χ^*2*^ = 0.0, *df* = 1, *P* = 1.0) and 75% in Cotonou (χ^*2*^ = 2.200, *df* = 1, *P* = 0.1380).

**Table 3:** Nature and types of breeding sites in the different cities according to season

Cities	Rainy season					Dry season				
	Type of site		Nature of the site		Total	Type of site		Nature of the site		Total
	Temporary, *n* (%)	Permanent, *n* (%)	Natural, *n* (%)	Artificial, *n* (%)	*n* (%)	Temporary, *n* (%)	Permanent, *n* (%)	Natural, *n* (%)	Artificial, *n* (%)	*n* (%)
Parakou	35 (81.4)	8 (18.6)	17 (39.5)	26 (60.5)	43 (100)	5 (50)	5 (50)	2 (20)	8 (80)	10 (100)
Natitingou	30 (83.3)	6 (16.7)	21 (58.3)	15 (41.7)	36 (100)	3 (60)	2 (40)	2 (40)	3 (60)	5 (100)
Bohicon	39 (97.5)	1 (2.5)	28 (70)	12 (30)	40 (100)	8 (100)	0 (00)	1 (12.5)	7 (87.5)	8 (100)
Cotonou	42 (93.3)	3 (6.7)	35 (77.8)	10 (22.2)	45 (100)	9 (75)	3 (25)	3 (25)	9 (75)	12 (100)

The breeding sites were considered to be either natural or artificial. The natural sites included puddles, ponds and swamps. Habitats such as gutters, tyres, construction sites, tips, cans, and vegetable farms, which were manmade or resulted from human activity, were classified as artificial. In Natitingou, Bohicon and Cotonou, natural sites were more abundant than artificial ones during the rainy season, and accounted for 58.1%, 70% and 77.1% of sites, respectively. However, in Parakou, artificial sites (60.1%) were more numerous than natural sites (39.1%) during the same season. In the dry season, the cities surveyed had high proportions of artificial sites (Table 3).

### Physicochemical parameters and their effects on the larval productivity of the breeding sites

Tables 4 and 5 show the mean physicochemical characteristics of the different types of breeding sites in the rainy and dry season. During the rainy season, the highest (39.9 °C) and lowest (24.1 °C) temperatures were recorded in a pond and in a tyre, respectively, in Parakou. The pH ranged from 2.88 to 10.44. The lowest pH was recorded on a vegetable farm and the highest in a puddle during the rainy season in the city of Parakou. Dissolved oxygen was highest (8.23 mg/L) in a swamp in Natitingou and lowest in a gutter in Bohicon. The highest salinity (0.8 g/L) and conductivity (1435 μS/cm) were measured during the rainy season in tyre tracks at Parakou. The lowest salinity (0.1 g/L) was recorded in a large habitat while the lowest conductivity (43.2 μS/cm) was recorded in a tyre during the rainy season in Bohicon. The highest turbidity, 970 NTU, was recorded in a gutter, while the lowest turbidity, 1.35 NTU, was recorded in a tyre in Bohicon during the rainy season.

**Table 4:** Physicochemical parameters of the breeding sites in the rainy season

Breeding sites	*n*	Physicochemical parameters							Depth (m)	Area (m^2^)
		Temperature (°C)	pH	Dissolved oxygen (mg/L)	Conductivity(μS/cm)	Salinity (g/L)	TDS (p.p.m.)	Turbidity (NTU)		
Gutters	12	30.86 ± 3.14	7.13 ± 0.10	2.19 ± 1.12	536.11 ± 329.18	0.13 ± 1.16	388.16 ± 235.13	240.16 ± 338.18	0.16 ± 0.07	1.13 ± 0.10
Tyres	14	27.15 ± 2.18	7.15 ± 0.12	2.10 ± 1.15	401.12 ± 296.12	0.11 ± 0.18	247.13 ± 190.18	33.16 ± 34.16	0.07 ± 0.03	0.12 ± 0.109
Vegetable farms	4	31.12 ± 5.12	6.15 ± 2.10	2.19 ± 0.15	525.1 ± 420.11	0.15 ± 0.17	364.15 ± 299.11	20.17 ± 26.19	0.06 ± 0.03	0.15 ± 1.14
Puddles	78	31.15 ± 3.10	8.19 ± 0.18	2.10 ± 1.13	302.14 ± 218.14	0.13 ± 0.19	184.12 ± 144.16	179.12 ± 220.14	0.12 ± 0.13	3.18 ± 1.14
Water containers for animals	1	25.1	9.13	2.15	540	0.1	447.1	109	0.2	0.19
Hoof imprints	2	32.15 ± 8.19	7.12 ± 0.17	2.19 ± 2.17	475 ± 200.11	0.15 ± 0.17	295.1 ± 87.11	562.1 ± 118.18	0.1	0.14 ± 0.19
Tin cans	1	28.1	9.14	0.1	922	0.1	507	38.12	0.05	0.13
Ponds	12	30.15 ± 3.14	7.16 ± 0.15	3.19 ± 1.12	350.16 ± 205.12	0.16 ± 0.11	224.14 ± 142.17	113.17 ± 163.18	0.22 ± 0.20	2.17 ± 0.10
Tyre tracks	11	30.1 ± 3.12	7.17 ± 0.15	2.15 ± 1.17	602.1 ± 520.17	0.17 ± 0.19	416.13 ± 392.17	255.18 ± 246.19	0.08 ± 0.03	0.16 ± 0.16
Swamps	2	30.1 ± 5.13	7.17 ± 0.17	7.19 ± 0.19	373.1 ± 111.19	0.15 ± 0.17	232.15 ± 79.17	39.13 ± 22.19	0.15 ± 0	1.12 ± 0.19
Pits	3	30.1 ± 0.18	7.16 ± 0.16	3.15 ± 1.18	603.16 ± 329.19	0.13 ± 0.15	377.15 ± 217.18	9.17 ± 1.19	0.55 ± 0.42	2.18 ± 1.14
Construction sites	1	27.1	8.15	3.12	220.1	0.1	139.1	17.18	0.03	4.11

*NTU* Nephelometric turbidity units

**Table 5:** Physicochemical parameters of the breeding sites in the dry season

Breeding sites	*n*	Physicochemical parameters							Depth (m)	Area (m^2^)
		Temperature (°C)	pH	Dissolved oxygen (mg/L)	Conductivity (μS/cm)	Salinity (g/L)	TDS (p.p.m.)	Turbidity (NTU)		
Gutters	10	30.6 ± 2.12	8.10 ± 0.11	2.11 ± 1.1	583.1 ± 179.1	0.14 ± 0.17	203.1 ± 193.1	24.13 ± 4.13	0.11 ± 0.16	1.16 ± 1.16
Tyres	3	31.1 ± 2.14	8.15 ± 0.12	0.19 ± 1.1	285.18 ± 333.1	0.19 ± 0.11	182.1 ± 137.1	17.18 ± 13.10	0.06 ± 0.001	0.12 ± 0.107
Vegetable farms	5	29.1 ± 1.14	8.18 ± 0.19	2.15 ± 2.12	621.1 ± 290.1	0.12 ± 0.18	307.1 ± 261.1	10.10 ± 6.18	0.16 ± 0.16	2.18 ± 1.18
Puddles	6	31.1 ± 10.1	7.11 ± 0.18	1.10 ± 1.18	267.1 ± 214.1	0.12 ± 0.15	187.1 ± 98.1	106.10 ± 154.17	0.15 ± 0.07	2.15 ± 0.19
Tyre tracks	2	35.1 ± 1.13	7.15 ± 0.14	1.19 ± 1.15	791.1 ± 415.14	0.1 ± 0.17	316.1 ± 211.1	75.15 ± 18.11	0.08 ± 0.02	0.1 ± 0.12
Hollow bricks	1	31.1	6.19	2.14	333	0.1	233.1	36.16	0.10	0.15
Flowerpots	1	29.1	7.13	1.1	137.1	0.1	100.1	6.12	0.10	0.17
Under bridges	3	30.16 ± 1.10	7.11 ± 0.18	1.15 ± 1.11	523.1 ± 197.1	0.12 ± 0.11	404.1 ± 113.1	35.17 ± 20.19	0.50 ± 0.45	7.13 ± 0.10
Swamps	2	32.1 ± 0.11	7.11 ± 0.17	1.12 ± 0.19	453.1 ± 250.1	0.1 ± 0	185.1	195.16 ± 17.17	0.25 ± 0.07	12.16 ± 1.16
Pits	2	34.1 ± 3.11	7.18 ± 1.11	1.14 ± 0.11	381.1 ± 127.1	0.15 ± 0.17	243.1 ± 114.1	33.16 ± 26.11	0.25 ± 0.07	5.13 ± 7.13

**Fig. 4:**
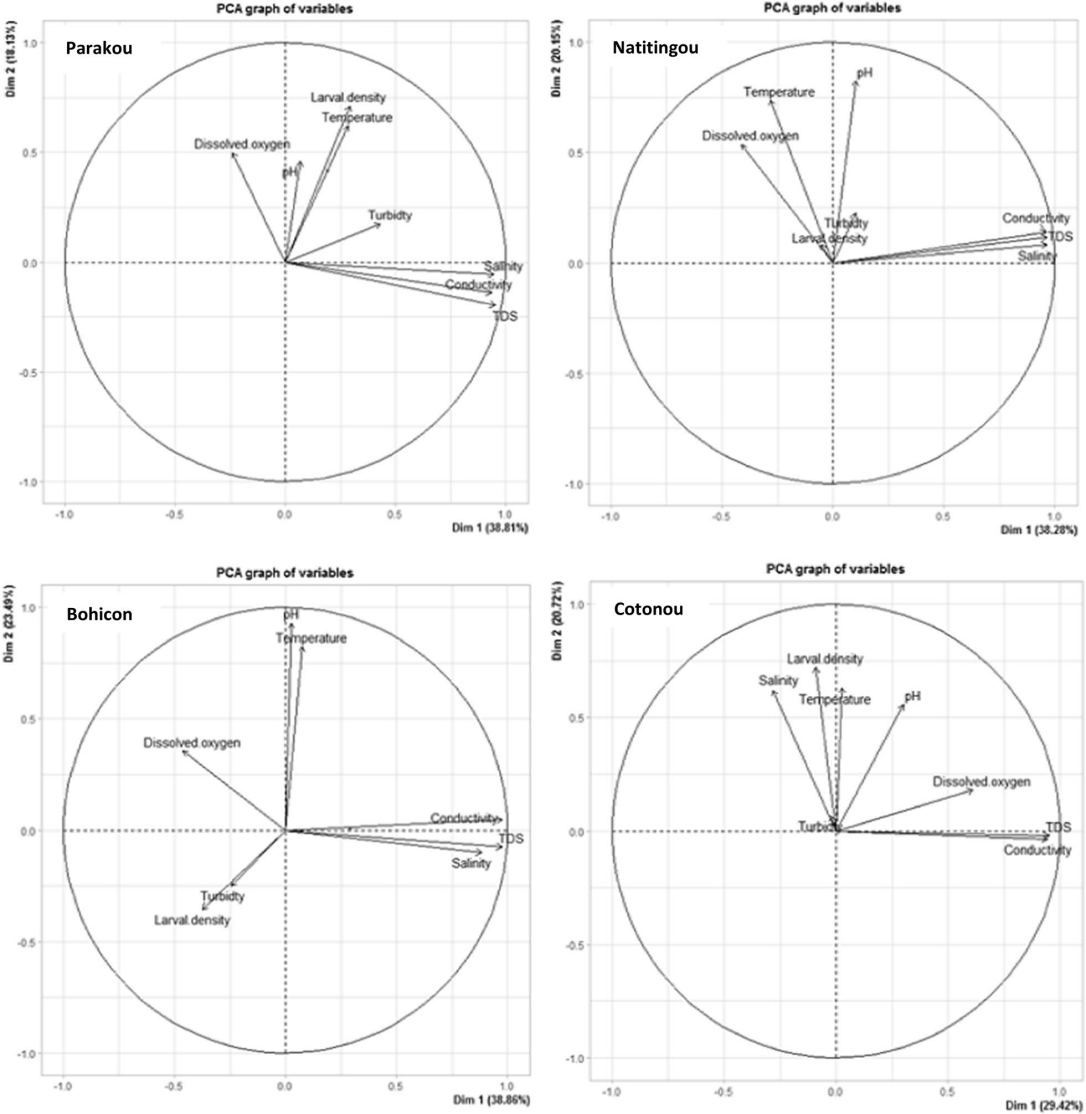
Projection of physicochemical parameters and larval density of *Anopheles* in the same plane [Dim1 × Dim2 of the principal component analysis (*PCA*)]

Principal component analysis was used to determine the relationships between the investigated physicochemical variables and *Anopheles* larval productivity. The projections showed that larval productivity was linked to the variation of some physicochemical parameters (Fig. 4). Indeed, in the city of Parakou, larval density was related to the 2nd PC axis and showed positive correlations with dissolved oxygen, pH and temperature. These positive correlations with dissolved oxygen (*r* = 0.26, *P* = 0.038) and temperature (*r* = 0.23 *P* = 0.047) were statistically significant. In the city of Natitingou, there was no significant correlation between any physicochemical parameter and the abundance of *Anopheles* larvae. In Bohicon, dissolved oxygen was related to larval density on the first PC axis, but this relationship was not statistically significant (*r* = 0.16, *P* = 0.862). In the city of Cotonou, temperature (*r* = 0.23, *P* = 0.048) and salinity (*r* = 0.44, *P* = 0.002) were positively correlated with the density of *Anopheles* larvae.

### Microbiological parameters and their effects on the larval productivity of the breeding sites

To determine whether bacterial composition could influence larval density, microbiological analysis of water samples from some of the habitats encountered in the study sites was performed. This allowed the isolation, identification and count of faecal coliforms, total coliforms and *E. coli* present in the samples. There were high loads of these bacteria in several water samples from the larval habitats. The average loads of faecal coliforms, total coliforms and *E. coli* were 2.18 × 10^5^ UFC/100 mL, 1.80 × 10^4^ UFC/100 mL, 2.20 × 10^4^ UFC/100 mL in Parakou; 6.60 × 10^5^ UFC/100 mL, 3.30 × 10^4^ UFC/100 mL, 9.40 × 10^4^ UFC/100 mL in Natitingou; 3.90 × 10^5^ UFC/100 mL, 4.40 × 10^4^ UFC/100 mL, 5.95 × 10^4^ UFC/100 mL in Bohicon; and 6.76 × 10^5^ UFC/100 mL, 9.90 × 10^4^ UFC/100 mL, 1.26 × 10^5^ UFC/100 mL in Cotonou, respectively.

**Fig. 5:**
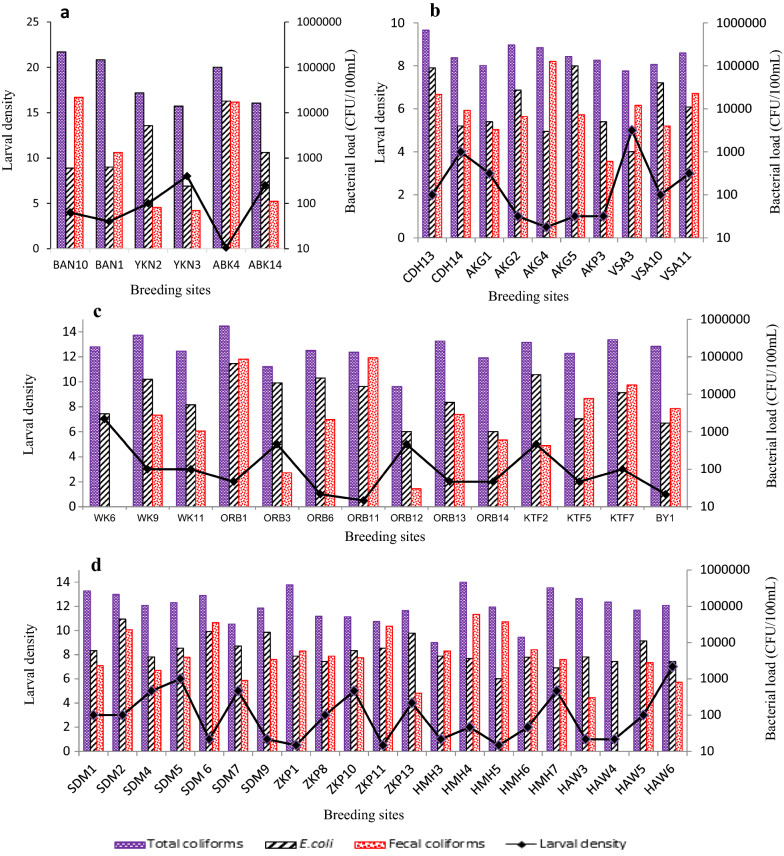
**a**–**d** Correlation of the microbiological parameters with the larval densities of the different breeding sites. **a** Parakou, **b** Cotonou, **c** Natitingou, **d** Bohicon

Figure 5 shows the results of the cross-sectional analysis between bacteriological load and larval densities of the water samples in the different cities. A significant negative correlation was observed between larval density and the concentration of coliforms (*r* = − 0.72, *P* = 0.0024). However, no significant correlation was observed between larval density of the breeding sites and total coliform load (*r* = − 0.22, *P* = 0.215) or *E. coli* load (*r* = 0.06, *P* = 0.419).

## Discussion

The present study was carried out to assess the spatiotemporal distribution of larval habitats as well as the physicochemical and microbiological variables favourable for *Anopheles* larval development in four major cities of Benin. Most of the *Anopheles* breeding sites encountered in the different districts resulted from human activities and their characteristics were related to factors associated with urbanization. Seventeen different types of larval habitats were identified, including puddles, abandoned tyres, construction sites and vegetable farms. A high diversity of *Anopheles* larval habitats was also reported by Djegbe et al. [34] for southern Benin, and by many other authors for Africa [35–38]. Puddles were the most numerous breeding sites recorded in the different study sites. These were generally found in alleys or on the edges of streets, and essentially indicated a lack of sanitation provided by public authorities and a poorly controlled urbanization process [39, 40]. In addition, the neglect of the local population and practices associated with economic activities could be responsible for the presence of many of the types of larval habitats [37, 41]. In Parakou, the abundance of tyres, which serve as larval habitats for *Anopheles*, could be due to increasing transportation by large trucks which serve other cities in the north and neighbouring countries, and transit the city. This leads to tyres being abandoned in the city’s many garages and tyre centres.

The classification of breeding sites as ‘natural’ or ‘artificial’ allowed us to highlight the role of humans in maintaining the transmission of malaria in the dry season through their routine activities. Indeed, though the number of larval habitats was considerably reduced during this season, those that were found were mainly man-made. Therefore, it is important to draw the attention of the population to their role in reducing the transmission of malaria through better sanitation and elimination of these habitats [38].

The larval habitats were also classified as ‘permanent’ or ‘temporary’. This classification of habitats helped us to understand their persistence and the extent to which they contributed to the abundance of *Anopheles* populations during seasonal changes in the study areas [42]. A high number of temporary habitat types were observed in the four cities during the two collection periods. As the temporary breeding sites depend mainly on rainfall, and dry out quite quickly with a drop in precipitation, the high proportions of them observed in the dry season could have been due to human activities. Several types of human activities that take place in the cities were found to create suitable oviposition sites in the dry season. In particular, the colonization of lowland areas for housing, agriculture, public works, or domestic and industrial activities was one of the important factors responsible for the creation of potential breeding sites for anopheline mosquitoes.

In three of the four cities surveyed, Natitingou, Bohicon and Cotonou, the majority of *Anopheles* breeding sites were found in urban districts. This could be explained by the fact that, in urban districts, breeding sites are accessible and easily spotted, unlike in districts with low urban potential [34]. In addition, urban districts offer a multiplicity of typical breeding sites (temporary, sunny and clear of debris) favourable to *Anopheles*, in contrast to non-urban districts, which are characterized by unsanitary conditions, and where potential breeding sites are often highly polluted and thus less favourable for *Anopheles* development [43, 44]. These results are interesting because they may explain the existence of a fairly high urban rate of transmission of malaria, which a few years ago was still considered controversial [45]. A high rate of malaria transmission in urban areas was recently reported by Nahum et al. [7], who found that Cotonou could classify as a hyperendemic area, with perennial malaria transmission, and spleen rates between 40 and 60%. Yadouleton et al. [46] also observed a high biting rate and sporozoïte infection of *Anopheles* in urban districts of Cotonou, Porto-Novo and Parakou.

In Cotonou and Bohicon, tyre tracks were the most productive habitat types. This can be explained by the fact that tyre tracks constitute ideal sites for oviposition by *Anopheles* females due to their small surface area, adequate exposure to sunlight and absence of surface plant debris [47, 48]. Other than tyre tracks, puddles, vegetable farms and gutters were among the habitat types with the highest larval densities in the four cities. Mattah et al*.* [39] reported similar results, with puddles (13.7 larvae/dip), gutters (12.9/dip) and urban farm sites (11.6/dip) the most productive larval habitats for *Anopheles* in southern Ghana.

It is also important to note that the larval abundances of the different types of habitats were mainly influenced by season and physicochemical parameters. There was seasonal variation in larval densities, with higher densities in the rainy season compared to the dry season in all types of breeding sites. It is therefore appropriate that the various control strategies used to reduce malaria transmission should be intensified during the dry seasons [49]. The physicochemical characterization of the habitats made it possible to identify positive correlations between the density of *Anopheles* larvae and certain parameters, including temperature, oxygen level and pH. A positive relationship between *Anopheles* larval density and temperature has been reported previously by several authors [50–52]. According to Muturi et al*.* [52], low water temperatures cause a decline in the growth of microorganisms on which mosquito larvae feed. In addition, higher temperatures can be detrimental to many aquatic arthropods, including predators of larvae, and thus increase the chance of survival of *Anopheles* larvae [18]. Higher temperatures may therefore favour the development of larval stages.

As in the present study, Mahamane et al*.* [9] and Mbidda et al*.* [53] found a positive correlation between *Anopheles* larval density and dissolved oxygen concentration. As *Anopheles* larvae do not have a siphon, dissolved oxygen in the water could theoretically be an important factor for their survival. Other authors have reported that anopheline larvae prefer fresh, well-oxygenated water with a low mineral content [24]. However, it should be noted that the *Anopheles* larvae showed a preference for water of higher salinity in the city of Cotonou. This positive correlation of the density of *Anopheles* larvae with salinity suggests an adaptation of this species to unusual breeding sites. The proximity of Cotonou, which is a coastal city, to the sea is believed to explain the, at times, high salt levels of the breeding sites. In addition, anthropogenic factors, which are more accentuated in Cotonou due to the large population and development of industrial activities there, could have an effect on the quality of water in the larval habitats. These observations agree with the findings of many authors [41, 54] who found that the *Anopheles* larvae in brackish roosts in Cape Coast (Ghana) were mainly of the species *Anopheles coluzzii*. Subsequent work, including molecular identification, will be carried out in order to analyse the distributions of the species of the *Anopheles gambiae* complex within the different types of breeding sites identified here.

The microbiological analysis indicated the presence of faecal and total coliforms at various concentrations in the water samples from the breeding sites. The presence of these bacteria indicates faecal contamination of the breeding sites, which may be of human or animal origin. Furthermore, a negative correlation was observed between the density of *Anopheles* larvae and the concentrations of faecal coliforms, which suggests that these coliforms inhibit the growth of the larvae [24]. While bacteria are known to be a source of nutrients for the growth of mosquito larvae, they may also be indicative of environmental conditions favourable to the presence, or absence, of larvae in breeding sites [55]. For example, Dada et al. [56] showed that the abundance of *E. coli* in breeding sites was significantly negatively correlated with the density of *Aedes aegypti* larvae. Experimental work in the laboratory will be carried out to better understand the mechanisms associated with the anti-larval activity of faecal coliforms.

The results of this study are important for the design of a larval control strategy for the cities of Benin. They indicated that the most productive *Anopheles* breeding sites were in specific man-made habitats. The findings of this study provide support for the consideration of environmental management for malaria control in urban settings. In terms of priorities, urban malaria is most efficiently controlled through highly focused, community-level interventions. The emphasis here should be on eliminating vector breeding sites through larvicidal and other measures. While LLINs and IRS are the gold standard for vector control in rural areas, there is great potential for the identification and hence elimination of mosquito breeding sites in urban settings by paying attention to both natural and artificial habitats.

## Conclusions

This study shows that many factors, such as climatic conditions and human activities, affect larval proliferation because they support the presence of artificial breeding sites throughout the year. The abundance of *Anopheles* larvae was positively associated with physicochemical determinants including high temperatures and dissolved oxygen levels, and negatively with faecal coliform concentrations, which likely inhibit larval growth. The present study provides a great deal of important information that should be taken into consideration by the National Malaria Control Program for the development of future malaria control strategies. Nowadays, vector control strategies cannot only rely on the promotion of LLINs and IRS, as is presently the case in Benin. Programmes that employ a combination of larvicidal treatments and environmental measures designed to reduce the number of breeding sites should be a central feature of urban malaria control.

## Data Availability

The data sets analysed during this study are available from the corresponding author on reasonable request.

## Abbreviations

IRS: Indoor residual spraying
LLINs: Long-lasting insecticidal nets
PCA: Principal component analysis
TDS: Total dissolved solids

## References

[1] Keiser J, Utzinger J, De Castro MC, Smith TA, Tanner M, Singer BH (2004). Urbanization in sub-Saharan Africa and implication for malaria control. Am J Trop Med Hyg.

[2] Mathanga DP, Tembo AK, Mzilahowa T, Bauleni A, Mtimaukenena K, Taylor TE (2016). Patterns and determinants of malaria risk in urban and peri-urban areas of Blantyre. Malawi Malar J.

[3] Kudom AA, Mensah BA, Agyeman TK (2012). Characterization of mosquito larval habitats and assessment of insecticide-resistance status of *Anopheles gambiae* senso lato in urban areas in southwestern Ghana. J Vector Ecol.

[4] Klinkenberg E, McCall PJ, Wilson MD, Amerasinghe FP, Donnelly MJ (2008). Impact of urban agriculture on malaria vectors in Accra, Ghana. Malar J.

[5] Matthys B, N’Goran EK, Koné M, Koudou BG, Vounatsou P, Cissé G (2006). Urban agricultural land use and characterization of mosquito larval habitats in a medium-sized town of Côte d’Ivoire. J Vector Ecol.

[6] Mourou J-R, Coffinet T, Jarjaval F, Cotteaux C, Pradines E, Godefroy L (2012). Malaria transmission in Libreville: results of a one-year survey. Malar J.

[7] Nahum A, Erhart A, Mayé A, Ahounou D, van Overmeir C, Menten J (2010). Malaria incidence and prevalence among children living in a peri-urban area on the coast of Benin, West Africa: a longitudinal study. Am J Trop Med Hyg.

[8] Fonds des Nations Unies pour l'Enfance, Afrique Génération 2030. Favoriser les investissements dans l’enfance pour bénéficier du dividende démographique. Résumé Exécutif. 2021.

[9] Mahamane Iro S, Seydou YA, Doumma A (2020). Mesures des indicateurs de prolifération des larves de moustiques au niveau des mares permanentes et semi-permanentes de Saga, Niger. Int J Biol Chem Sci.

[10] World malaria report 2018. Geneva: World Health Organisation. 2018. http://www.who.int/malaria/publications/world_malaria_report_2018/report/en/. Accessed 25 Apr 2021.

[11] Akogbeto M, Padonou GG, Bankole HS, Gazard DK, Gbedjissi GL (2011). Dramatic decrease in malaria transmission after large-scale indoor residual spraying with bendiocarb in Benin, an area of high resistance of *Anopheles gambiae* to pyrethroids. Am J Trop Med Hyg.

[12] Ossè R, Aikpon R, Padonou GG, Oussou O, Yadouléton A, Akogbéto M (2012). Evaluation of the efficacy of bendiocarb in indoor residual spraying against pyrethroid resistant malaria vectors in Benin: results of the third campaign. Parasit Vectors.

[13] Rowland M, Boko P, Odjo A, Asidi A, Akogbeto M, N’Guessan R (2013). A new long-lasting indoor residual formulation of the organophosphate insecticide pirimiphos-methyl for prolonged control of pyrethroid-resistant mosquitoes: an experimental hut trial in Benin. PLoS ONE.

[14] Akoton R, Tchigossou GM, Djegbe I, Yessoufou A, Atoyebi SM, Tossou E (2018). Experimental huts trial of the efficacy of pyrethroids/piperonyl butoxide (PBO) nets treatments for controlling multi-resistant populations of *Anopheles funestus s*.s. in Kpomè, Southern Benin. Wellcome Open Res.

[15] Djouaka R, Riveron JM, Yessoufou A, Tchigossou G, Akoton R, Irving H (2016). Multiple insecticide resistance in an infected population of the malaria vector *Anopheles funestus* in Benin. Parasit Vectors.

[16] Djegbe I, Missihoun A, Djouaka R, Akogbeto M (2017). Surveillance entomologique: dynamique de la population et de la résistance aux insecticides chez *Anopheles gambiae*
*s.l* en milieu de riziculture irriguée au Sud Bénin. J Appl Biosci.

[17] World Health Organization. World malaria report 2019. Geneva: World Health Organization. 2019. http://www.who.int/malaria/fr. Accessed 28 Apr 2021.

[18] Soleimani-Ahmadi M, Vatandoost H, Zare M (2014). Characterization of larval habitats for anopheline mosquitoes in a malarious area under elimination program in the southeast of Iran. Asian Pac J Trop Biomed.

[19] Floore TG (2006). Mosquito larval control practices: past and present. J Am Mosq Control Assoc.

[20] WHO (2016). World malaria report 2016.

[21] Nikookar SH, Fazeli-Dinan M, Azari-Hamidian S, Mousavinasab SN, Aarabi M, Ziapour SP (2017). Correlation between mosquito larval density and their habitat physicochemical characteristics in Mazandaran Province, northern Iran. PLoS Negl Trop Dis.

[22] Wang H, Wang Y, Cheng P, Wang H, Wang H, Liu H (2021). The larval density of mosquitos (Diptera: Culicidae) in Jiaxiang County, Shandong Province, China: influence of bacterial diversity, richness, and physicochemical factors. Front Ecol Evol.

[23] Omolade OO, Adetutu SA (2018). Oviposition and breeding water sites preferences of mosquitoes within Ojo area, Lagos State, Nigeria. Biomed J Sci Tech Res.

[24] El Ouali Lalami A, El hilali O, Benlamlih M, Merzouki M, Raiss N, Ibensouda Koraichi S (2010). Etude entomologique, physicochimique et bactériologique des gîtes larvaires de localités à risque potentiel pour le paludisme dans la ville de Fès. Bull Inst Sci Rabat.

[25] INSAE (2016). Effectifs de la population des villages et quartiers de ville du Benin (RGPH-4, 2013).

[26] Akomagni L. Monographie de la commune de Cotonou. Programme d’appui au démarrage des communes. Afrique Conseil. 2006.

[27] Houngnihin R. Monographie de la commune de Bohicon. Programme d’appui au démarrage des communes. Afrique Conseil. 2006.

[28] Kora O. Monographie de la commune de Parakou. Programme ‘appui au démarrage des communes. Afrique Conseil. 2006.

[29] Biaou CF. Monographie de la commune de Natitingou. Programme d’appui au démarrage des communes. Afrique Conseil. 2006.

[30] Silver JB, Service MW (2007). Mosquito ecology: field sampling methods.

[31] Talipouo A, Ntonga-Akono P, Tagne D, Mbida-Mbida A, Etang J, Tchoffo-Fobasso R (2017). Comparative study of Culicidae biodiversity of Manoka island and Youpwe mainland area, littoral, Cameroon. Int J of Biosci.

[32] Gillies MT, de Meillon B (1968). The Anophelinae of Africa south of the Sahara; Ethiopian zoogeographical region.

[33] Nonfodji OM, Fatombi JK, Ahoyo TA, Boya B, Baba-Moussa LS, Aminou T (2020). Effect of KMnO_4_ amounts on antibacterial property of activated carbon for efficient treatment of northern Benin hospital wastewater in a fixed bed column system. Int J Hyg Environ Health.

[34] Djègbè I, Toponon F, Gbankoto A, Tchigossou G, Djossou-Hessou D, Dossou C (2019). Typologie des gîtes larvaires et résistance des vecteurs du paludisme à la deltaméthrine dans les milieux urbain et rural du département de l’atlantique au sud du Benin: données préliminaires. Euro Sci J.

[35] Koumba AA, Zinga Koumba CR, Mintsa Nguema R, Djogbenou LS, Obame Ondo P, Ketoh GK (2018). Distribution spatiale et saisonnière des gîtes larvaires des moustiques dans les espaces agricoles de la zone de Mouila, Gabon. Int J Biol Chem Sci.

[36] Ntonga-Akono P, Mbida Mbida A, Awono AP, Youmbi EL, Abdel Kayoum Y, Kekeunou S (2018). Habitats larvaires et sensibilité des vecteurs du paludisme aux insecticides dans des localités (semi-urbaine et rurale) de la région du littoral Camerounais: données préliminaires. Rev Ecol.

[37] Tia E, Gbalégba NGC, M’Bra KR, Kaba A, Boby OAM, Koné M (2016). Étude du niveau de production larvaire d’*Anopheles gambiae* s.l. (Diptera: Culicidae) dans différents types de gîtes à Oussou-yaokro au Centre-Ouest et à Korhogo, au Nord (Côte d’Ivoire). J Appl Biosci.

[38] Hinne I, Attah SA, Mensah BA, Forson AO, Afrane YA (2021). Larval habitat diversity and *Anopheles* mosquito species distribution in different ecological zones in Ghana. Parasit Vectors.

[39] Mattah P, Dzorgbe A, Godfred F, Amekudzi LK, Mattah MM, de Souza DK (2017). Diversity in breeding sites and distribution of *Anopheles* mosquitoes in selected urban areas of southern Ghana. Parasit Vectors.

[40] Bouabida H, Djebbar F, Soltani N (2012). Etude systématique et écologique des moustiques (Diptera: Culicidae) dans la région de Tébessa (Algérie). Faun Entomol.

[41] Kudom AA (2015). Larval ecology of *Anopheles coluzzii* in Cape Coast, Ghana: water quality, nature of habitat and implication for larval control. Malar J.

[42] Imbahale SS, Paaijmans KP, Mukabana WR, Lammeren RV, Githeko AK, Takken W (2011). A longitudinal study on *Anopheles* mosquito larval abundance in distinct geographical and environmental settings in western Kenya. Malar J.

[43] Ajayi MB, Adeleke MA, Idowu ET, Awolola TS (2010). Surveillance of mosquitoes vectors in Ajumoni Estate Ogun State. Nigeria An Biol Res.

[44] Gimnig JE, Ombok M, Kamau L, Hawley WA (2001). Characteristics of larval anopheline (Diptera: Culicidae) habitats in western Kenya. J Med Entomol.

[45] Fournet F, Kassié D, Dabiré RK, Salem G (2015). Analyse de la distribution socio-spatiale du paludisme dans une ville moyenne ouest africaine, Bobo-Dioulasso (Burkina Faso). J Int Geosci Environ.

[46] Yadouléton A, N'Guessan R, Allagbé H, Asidi A, Boko M, Osse R (2010). The impact of the expansion of urban vegetable farming on malaria transmission in major cities of Benin. Parasit Vectors.

[47] Antonio-Nkondjio C, Tene Fossog B, Ndo C, Menze DB, Zebaze TS, Awono-Ambene P (2011). *Anopheles gambiae* distribution and insecticide resistance in the cities of Douala and Yaoundé (Cameroon): influence of urban agriculture and pollution. Malar J.

[48] Sy O, Konaté L, Ndiaye A, Dia I, Diallo A, Taïrou F (2016). Identification des gîtes larvaires d’anophèles dans les foyers résiduels de faible transmission du paludisme “hotspots” au centre-ouest du Sénégal. Bul Soc Pathol Exot.

[49] Mala AO, Irungu LW, Shililu JI, Muturi EJ, Mbogo CM, Njagi JK (2011). *Plasmodium falciparum* transmission and aridity: a Kenyan experience from the dry lands of Baringo and its implications for *Anopheles arabiensis* control. Malar J.

[50] Benhissen S, Habbachi W, Rebbas K, Masna F (2018). Études entomologique et typologique des gîtes larvaires des moustiques (Diptera: Culicidae) dans la région de Bousaâda (Algérie). Bull Soc R Sci Liège.

[51] Onchuru TO, Ajamma YU, Burugu M, Kaltenpoth M, Masiga D, Villinger J (2016). Chemical parameters and bacterial communities associated with larval habitats of * Anopheles*,* Culex* and* Aedes* mosquitoes (Diptera: Culicidae) in western Kenya. Int J Trop Insect Sci.

[52] Muturi EJ, Mwangangi J, Shililu J, Jacob BG, Mbogo C, Githure J (2008). Environmental factors associated with the distribution of *Anopheles arabiensis* and *Culex quinquefasciatus* in a rice agro-ecosystem in Mwea, Kenya. J Vector Ecol.

[53] Mbida Mbida A, Etang J, Akono-Ntonga P, Eboumbou MC, Awono-Ambene P, Tagne D (2017). Nouvel aperçu sur l’écologie larvaire d’*Anopheles coluzzii* Coetzee et Wilkerson, 2013 dans l’estuaire du Wouri, littoral-Cameroun. Bull Soc Pathol Exot.

[54] McLaughlin K, Burkot TR, Oscar J, Beebe NW, Russell TL (2019). Defining the larval habitat: abiotic and biotic parameters associated with *Anopheles farauti* productivity. Malar J.

[55] Nilsson LKJ, Sharma A, Bhatnagar RK, Bertilsson S, Terenius O (2018). Presence of *Aedes* and *Anopheles* mosquito larvae is correlated to bacteria found in domestic water-storage containers. FEMS Microbiol Ecol.

[56] Dada N, Jumas-Bilak E, Manguin S (2014). Comparative assessment of the bacterial communities associated with *Aedes aegypti* larvae and water from domestic water storage containers. Parasit Vectors.

